# Bran-Enriched Fractions from Blue and Purple Wheat Improve Antioxidant Potential and Nutritional Profile

**DOI:** 10.3390/foods15091598

**Published:** 2026-05-05

**Authors:** Samuela Palombieri, Giuliana Bruno, Maria Dolores Garcia Molina, Alessandro Cammerata, Cecilia Miccoli, Linda Felici, Sara Francesconi, Gianluca Giuberti, Federica Castellani, Matteo Vitali, Giorgio Mariano Balestra, Francesco Sestili

**Affiliations:** 1 Department of Agriculture and Forest Sciences (DAFNE), University of Tuscia, Via San Camillo de Lellis snc, 01100 Viterbo, Italy; palombieri@unitus.it (S.P.); giuliana.bruno@unitus.it (G.B.); mariadolores.garcia@unitus.it (M.D.G.M.); cecilia.miccoli@unitus.it (C.M.); felici@unitus.it (L.F.); francesconi.s@unitus.it (S.F.); 2 Council for Agricultural Research and Economics, Research Centre for Engineering and Agro-Food Processing, Via Manziana 30, 00189 Rome, Italy; alessandro.cammerata@crea.gov.it; 3 Department for Sustainable Food Process, Università Cattolica del Sacro Cuore, 29122 Piacenza, Italy; gianluca.giuberti@unicatt.it; 4 Department of Public Health and Infectious Diseases, University of Rome La Sapienza, 00185 Rome, Italy; federica.castellani@uniroma1.it (F.C.); matteo.vitali@uniroma1.it (M.V.)

**Keywords:** pigmented wheat, anthocyanins, functional foods, micronization, air classification, antioxidant capacity

## Abstract

Pigmented wheat varieties represent a promising source of bioactive compounds, particularly anthocyanins, with potential applications in the development of functional cereal-based foods. This study investigated the combined effect of pigmented wheat genetics and innovative milling technologies on the nutritional and technological properties of wheat-derived products. Two pigmented bread wheat genotypes, the blue-grained cultivar Purendo and the purple-grained line Vanilnoir, were compared with the non-pigmented cultivar Peralba. Grains were processed using conventional milling or through micronization followed by air-classification to obtain enriched fractions (F250 and G250). The resulting flours and fractions were evaluated for compositional traits, rheological properties, antioxidant activity, and pasta-making performance. Air-classification significantly increased ash, protein, and lipid contents while reducing total starch, confirming the enrichment of outer kernel components. Bran-enriched fractions exhibited enhanced antioxidant capacity, with the highest FRAP and TEAC values observed in pigmented genotypes. Pasta produced from enriched fractions showed improved nutritional profiles and, in most cases, a reduced predicted glycemic index compared with conventional flour-based pasta. Technological responses were genotype-dependent: while bran enrichment negatively affected dough rheology, the purple genotype maintained more balanced technological and sensory properties in pasta compared with the blue genotype. These results demonstrate that integrating pigmented wheat genetics with targeted milling strategies can support the development of functional cereal-based foods with enhanced antioxidant potential and improved nutritional quality.

## 1. Introduction

Bread wheat (*Triticum aestivum* L.) is one of the world’s most economically important crops and a staple food for millions of people. Through a wide range of traditional products, it provides a major share of global caloric and protein intake. Historically, wheat breeding programs have focused primarily on yield and technological quality traits, including test weight, grain hardness, protein content, and gluten strength. However, increasing consumer awareness of the relationship between diet and health has shifted research interest toward improving the nutritional and functional quality of wheat-based foods. This trend has stimulated efforts to enhance both the concentration and bioavailability of essential nutrients, such as minerals, vitamins, dietary fibers (notably arabinoxylans and resistant starch), and phenolic compounds [1,2,3,4,5]. Among the strategies explored to improve the nutritional value of wheat products, the exploitation of pigmented wheat genotypes has received increasing attention. Wheat’s versatility in end-use applications and its longer shelf life compared with many other bioactive-rich foods make it an excellent target for biofortification strategies aimed at improving human nutrition. Among the nutritional components of particular interest are anthocyanins, flavonoid pigments recognized for their strong antioxidant activity and potential to mitigate oxidative stress and reduce chronic disease risk [6]. Although typically associated with fruits and vegetables, anthocyanins are also found in pigmented wheat varieties, including those with purple or blue grains [7].

Purple grain pigmentation in wheat is associated with the accumulation of anthocyanins in the outer layers of the kernel, particularly in the pericarp. This trait was originally introduced into bread wheat from tetraploid wheat relatives such as *Triticum turgidum* subsp. *abyssinicum* and is now present in several wheat species showing different intensities of purple coloration [8,9,10]. In contrast, blue-grained wheat accumulates anthocyanins mainly in the aleurone layer. These pigments are controlled by distinct genetic factors and originate from different ancestral sources introgressed into modern wheat germplasm [11,12,13]. Blue and purple wheats differ not only in the location of anthocyanin accumulation but also in pigment composition and concentration [14]. Blue wheat typically contains a higher total anthocyanin content than its purple grain counterpart [15]. The dominant anthocyanins in blue grains are delphinidin 3-glucoside and delphinidin 3-rutinoside, whereas purple grains mainly contain cyanidin 3-glucoside and cyanidin 3-rutinoside [16]. In wheat, these pigments are concentrated in the bran and germ, parts that are often removed during the refining process. By enriching wheat-based products with bran fractions from pigmented genotypes, it is possible to retain a higher concentration of anthocyanins and other bioactive compounds, significantly enhancing their health benefits. Notably, anthocyanin levels have been reported to reach 157 mg/kg in wholemeal flour and 458 mg/kg in bran [17], highlighting the potential of bran fractions as a concentrated source of these beneficial compounds.

Both in vitro and in vivo studies, including clinical trials, have shown that whole-grain-derived anthocyanins can bolster antioxidant defenses, reduce oxidative stress, and contribute to the prevention of degenerative diseases [18]. Compared to more perishable sources like vegetables and fruits, wheat offers greater storage stability, making it a practical and scalable vehicle for delivering these health-promoting compounds. Pigmented cereals such as maize and rice are already incorporated into a range of food products, including tortillas and baby foods [19]. In contrast, the use of pigmented wheat in the food industry remains limited, largely because it is typically processed as whole flour.

Despite the recognized health benefits of whole wheat, its consumption is often hampered by sensory drawbacks. Wheat bran negatively affects dough properties by binding water through hydroxyl groups, reducing gluten development and compromising dough stability [20]. These factors impact the texture, volume, and overall acceptability of whole wheat products.

Emerging milling technologies such as micronization and air-classification offer promising solutions. Micronization pulverizes grains into finer particles, while air-classification separates them based on weight and size, enabling the isolation of enriched fractions [20,21]. These processes allow for the selective concentration of nutritionally valuable components located in the external layers of the kernel while maintaining acceptable technological performance of the derived flours.

In this study, the purple wheat genotype Vanilnoir and the blue wheat genotype Purendo were selected for their potential in biofortified pasta production. Using micronization and air-classification, we incorporated enriched bran fractions into pasta formulations, aiming to boost anthocyanin content while maintaining desirable sensory and textural qualities. This integrated approach highlights how the combination of pigmented wheat genetics and advanced milling technologies can contribute to the development of nutritionally improved pasta products with enhanced antioxidant potential.

## 2. Materials and Methods

### 2.1. Plant Material

Two bread wheat genotypes, one blue cultivar (Purendo) and one purple breeding line (Vanilnoir), along with a control non-pigmented genotype (Peralba) were used in this study (Table S1). Peralba is a soft wheat cultivar valued for its combination of grain quality, agronomic traits, and suitability for breadmaking. It was used as control in the evaluation of rheological properties and pasta quality. The three wheat genotypes were grown during the 2021–2022 season at the Experimental Farm “Nelo Lupori” of the University of Tuscia, located in Viterbo, Italy.

### 2.2. Arabinoxylan, β-Glucan and Amylose Content Determination

Seeds from the pigmented wheat genotypes Vanilnoir and Purendo, along with the control line Peralba, were ground using a Cyclotec mill (Foss Tecator AB, Hoganas, Sweden) equipped with a 0.5-mm sieve and analyzed for their fiber content (arabinoxylans and β-glucan). For each genotype, analyses were performed on a bulked flour sample, with three technical replicates. Total arabinoxylans (TOT-AX) and water-extractable arabinoxylans (WE-AX) were quantified in whole flours using colorimetric assays.

The method was based on Douglas [22] for TOT-AX with the adjustments outlined by Palombieri et al. [23]. Specifically, 10 mg of whole flour was mixed with 4 mL of distilled water and 20 mL of an extraction solution composed of 110 mL of glacial acetic acid, 2 mL of concentrated hydrochloric acid, 5 mL of 20% (*w*/*v*) phloroglucinol in ethanol, and 1 mL of 1.75% (*w*/*v*) glucose in water. The mixture was incubated in a boiling water bath for 25 min, with vortexing performed twice during this period. After incubation, the samples were cooled on ice for 5 min. Xylose content was determined by calculating the difference between absorbance readings at 552 nm and 510 nm. A standard curve for xylose was generated following the same procedure.

WE-AX was determined according to the protocol described by Finnie et al. [24]. The water-extractable fraction was obtained by mixing 125 mg of whole flour with 25 mL of distilled water. The resulting suspension was stirred for 30 min and then centrifuged at 2500 rpm for 10 min. For the determination of WE-AX, 1 mL of the supernatant was combined with 1 mL of distilled water and 10 mL of extraction solution, following the same procedure described above for TOT-AX. The concentrations of TOT-AX and WE-AX were calculated as a percentage of xylose using the specific equations reported in Palombieri et al. [24].

β-glucan concentration was measured in whole flour using the Mixed Linkage β-Glucan Assay Kit (K-BGLU, Megazyme Inc., Bray, Ireland).

Starch granules were extracted from a flour sample following Zhao and Sharp [25] with minor modifications. Ground seeds were soaked overnight in 1 mL of distilled water (dH_2_O) at 4 °C, vortexed, and centrifuged at 13,000 rpm for 5 min. The supernatant was discarded, and the pellet was mixed with 300 µL of dH_2_O to form gluten. The starch suspension was transferred to tubes containing 1 mL of 5 M NaCl and centrifuged. This washing step was repeated twice, followed by three washes with dH_2_O. After each wash, samples were vortexed, centrifuged, and the supernatant discarded. The final pellet was washed with 1 mL of acetone, centrifuged, air-dried for one hour, and stored at 4 °C.

Amylose content was determined using a colorimetric assay based on the iodine–amylose reaction [26]. Purified starch (15 mg) was dissolved in 2 mL of 1 M NaOH and 4 mL of dH_2_O, incubated in a boiling water bath for 30 min, and vortexed periodically to prevent gel formation. After cooling, 100 µL of the starch solution was mixed with 5 mL of 0.5% TCA and 50 µL of 0.01 N KI/I2, then incubated at room temperature for 20 min. Absorbance was measured at 610 nm.

A standard curve was prepared using potato amylose and maize amylopectin. Each value represents the mean ± standard error of six technical replicates per sample.

### 2.3. Protein Content

The protein content was determined using the Dumas method (ICC 167) [27] with a LECO FP528 device and Near-Infrared Transmission (NIT). Test weight was measured using (NIT). Ash content was assessed following the ISO 2171 [28] method at a temperature of 570 °C ± 10 °C in two repetitions. The dry gluten content was evaluated according to the EN ISO 21415 method [29]. The gluten index was analyzed using the automatic Glutomatic System (PerkinElmer, Springfield, IL, USA) and according to the ICC 158 method [30]. Kernel hardness was assessed using the Perten SKCS (Perten Instrument AB, Huddinge, Sweden), following the procedure outlined in the Single Kernel Characterization System 4100 Manual [31]. Three technical replicates were performed for each analysis.

### 2.4. Sedimentation Test

The sedimentation test was performed following the protocol described by Axford et al. [32], with two technical replicates for each sample. The analysis was conducted using 1 g of wholemeal flour obtained from a pooled seed sample.

Each sample was placed in a Pyrex glass tube with a hermetic seal. Four milliliters of distilled water were added, and the tubes were shaken horizontally 10 times, vortexed for 10 s, and left to rest for 5 min. After a brief 2-s vortex, the samples were allowed to settle for another 5 min. Subsequently, 12 mL of reaction buffer, consisting of 2% SDS and 0.18% lactic acid, was added. The tubes were shaken horizontally 10 times and left to rest for 20 min before measuring the height of the precipitated sediment.

### 2.5. Milling Procedures

Each bread wheat sample (Vanilnoir, Purendo, and Peralba) was divided into two portions, processed using different milling techniques. One portion underwent traditional milling to produce refined flour, while the other was processed with an advanced method involving micronization and air classification to obtain semi-wholemeal flour.

For the conventional milling process, the grains were conditioned at 17% moisture for approximately 20 h before being processed in a pilot milling system (Namad, Italy). The milling process yielded different fractions, including flour, bran, and fine bran, which were kept separate.

The second portion of each sample was micronized using a KMXi-300-7.5 unit (Separ Microsystem, Brescia, Italy), which operates with a steel drum containing a rotor spinning at a peripheral speed of around 170 Hz. A 0.7 mm sieve attached to the rotor controlled particle size. The micronization process achieved a yield of 97–98%.

Following micronization, the flour underwent air classification in an SX-500 apparatus (Separ Microsystem, Brescia, Italy) managed by a Mini-PLC system. The classification, controlled through a progressive opening valve, was performed at an air inlet setting of 250, resulting in two fractions: a coarse fraction (G250) and a fine fraction (F250).

### 2.6. Rheological Characterization of Different Flours

Flour and coarse fraction samples underwent a comprehensive quality assessment, including the determination of ash content, dry gluten, Gluten Index, and rheological properties through Alveograph and Farinograph tests. Each analysis was performed with a least two replicates per sample.

Ash content, dry gluten, and Gluten Index were measured as described in Section 2.3, with all tests conducted in duplicate. The Alveograph test was performed using a Chopin Alveograph (model MA82, equipped with an Alveolink NG recorder-computer) (Villeneuve-La-Garenne Cedex, Paris, France) following the AACC Method 54-30.02 [33] and UNI 10453 standards [34].

To assess dough behavior, the Farinograph test was carried out to evaluate water absorption and dough consistency, following ISO 530-1 guidelines [35]. The Falling Number was determined according to ICC Method 107/1 [36] to assess the enzymatic activity related to starch degradation.

Additionally, the yellow pigmentation of the samples was analyzed using a Minolta Chromameter CR 400 reflectance colorimeter equipped with a D65 illuminant and a specialized sample holder for granular materials (Minolta Italia, S.p.A., Milan, Italy).

### 2.7. Pasta Production and Colour Measurement

Flour and the F250 and G250 air-classified fractions from each genotype were used to produce spaghetti-shaped pasta with a diameter of 1.70 mm. The dough preparation and extrusion processes were conducted using an Italpast (Italpast S.r.l., Fidenza, Parma, Italy) experimental press, which features dual tanks, one designated for mixing and the other for kneading under vacuum conditions. The extrusion process was performed at a pressure ranging from 100 to 110 BAR. Hydration levels were adjusted to 32% for flour-based dough and 33% for the coarse fraction. A Teflon die was used for extrusion to shape the spaghetti. The freshly extruded pasta was then subjected to a drying process in a static drying chamber (Italpast S.r.l., Fidenza, Parma, Italy) equipped with a climate-controlled system managed via a PLC panel that regulates temperature and humidity throughout the drying cycle. A low-temperature drying protocol (50 °C for 21 h) was employed, after which the pasta samples were left to stabilize for approximately 10 days before undergoing quality evaluations.

The color of three distinct pooled pasta samples was measured in triplicate (three technical replicates per sample) using a CR400 colorimeter (Minolta Italia, S.p.A., Milan, Italy) equipped with a D65 illuminant and an 8 mm aperture. The instrument was calibrated before each session using a standard white calibration plate. Measurements were carried out on dry spaghetti samples that were previously equilibrated at room temperature (20 ± 1 °C) and arranged to form a uniform layer fully covering the measurement area, in order to avoid interference from gaps or surface irregularities. The assessment followed the CIE Lab* colour space (Commission Internationale de l’Éclairage), where L* indicates lightness (from 0 = black to 100 = white), a* represents the green (–) to red (+) axis, and b* the blue (–) to yellow (+) axis.

### 2.8. Evaluation of Pasta Cooking Quality

The cooking performance of spaghetti was evaluated following Quagliata et al. [37]. Briefly, 100 g of pasta was boiled in 1 L of unsalted tap water, with a strand removed every 30 s after 7 min to determine the Optimum Cooking Time (OCT) based on core disappearance. After draining, the spaghetti rested for 9 min before sensory analysis. A panel of five trained experts assessed firmness, stickiness, and bulkiness, scoring each from 10 to 100. The final quality score was the arithmetic mean of these attributes.

### 2.9. Pasta Nutritional Characterization

Raw spaghetti samples of *Peralba*, *Purendo*, and *Vanilnoir*, obtained from both flour and F250 air-classified fractions, were ground to a 1 mm particle size using a laboratory mill prior to analysis, with the exception of protein determination. All analyses were performed in technical duplicates. Moisture content was determined according to AOAC method 925.10 [38]. Briefly, 5 g of each sample was weighed into pre-dried and pre-weighed ceramic crucibles and dried in a ventilated oven at 105 °C for 24 h. Moisture content was calculated as the weight loss after drying. Ash content was determined following AOAC method 923.03 [39]. The dried samples were incinerated in a muffle furnace at 550 ± 10 °C until complete combustion was achieved. After cooling in a desiccator to room temperature, the crucibles were weighed, and ash content was calculated gravimetrically.

#### 2.9.1. Crude Lipids and Protein Content and Total Starch

Crude lipid content was determined according to AOAC 920.39 [40] using Soxhlet extraction. Briefly, 5 g of sample was extracted with petroleum ether, and lipid content was calculated gravimetrically after solvent removal and drying at 105 °C.

Crude protein content was determined following AOAC 2001.11 (Kjeldahl method) [41]. Samples were digested with sulfuric acid, distilled after alkalization, and the released ammonia was titrated. Protein content was calculated using a nitrogen-to-protein conversion factor of 6.25.

Total starch content was quantified using an enzyme assay kit (Megazyme assay kit K-TSTA 07/11, Megazyme International, Wicklow, Ireland) following the manufacturer’s instructions. Three technical replicates (repeated measurements of the same sample) were performed.

#### 2.9.2. Cooking Quality

The optimal cooking time (OCT) for spaghetti was determined using the AACC-approved method 66-50 [42]. OCT was defined as the time required for the white core to disappear when the spaghetti strand was compressed between two glass plates. Cooking loss (CL) was assessed by evaporating the cooking water to dryness overnight at 105 °C in an air oven, following the same AACC method. The remaining residue was weighed and expressed as a percentage of the initial spaghetti sample weight before cooking. To evaluate the degree of spaghetti hydration, water absorption capacity (WAC) was measured according to the AACC 66-50 method [42]. Ten grams of each sample were cooked to their optimal time in 100 mL of boiling distilled water, rinsed with cold water, drained for 30 s, and then weighed. The WAC was calculated as the weight difference before and after cooking. All analyses were performed in duplicate for each spaghetti batch.

#### 2.9.3. In Vitro Starch Digestion

A 10 g sample of spaghetti was cooked to the optimal time in 100 mL of boiling water, drained for 1 min, and then cut into small pieces to simulate mastication. Three grams of the sample were subjected to in vitro digestion in duplicate. The in vitro digestion was performed according to the method described by Minekus et al. [43], with some modifications. The oral phase (5 min/37 °C/180 rpm) was simulated by adding 5 mL of Simulated Salivary Fluid (SSF), consisting of 2 mM NaCl, 2 mM KCl, 24.7 mM NaHCO_3_, 6.67 mM urea, and α-amylase (about 7000 U/mL; Sigma A3176-1MU, Sigma–Aldrich^®^, Milan, Italy), with the pH adjusted to 6.8 [44]. The gastric phase (30 min/37 °C/180 rpm) was conducted by adding 5 mL of Simulated Gastric Fluid (SGF), a solution of 50 mM HCl (Sigma P-7000, Sigma–Aldrich^®^, Milan, Italy) containing 5 mg/mL of pepsin (Sigma P-7000, Sigma–Aldrich^®^, Milan, Italy), under horizontal agitation. Afterward, 20 mL of 100 mM sodium acetate buffer (0.1 M CH_3_COONa·3H_2_O, pH 5.2) was added to each sample at time 0 (T0), before proceeding to the intestinal phase (180 min/37 °C/180 rpm). The intestinal phase involved the addition of 5 mL of Simulated Intestinal Fluid (SIF), composed of amyloglucosidase (about 300 U/mL; Sigma A-7095, Sigma–Aldrich^®^, Milan, Italy), pancreatin (approximately 7500 FIP-U/g; Merck 7130, Merck KGaA, Darmstadt, Germany), and invertase (about 300 U/g; Sigma I-4504, Sigma–Aldrich^®^, Milan, Italy), at time points 30, 60, 120, and 180 min (T30′, T60′, T120′, and T180′, respectively). At each time point (T0, T30, T60, T120, and T180), 50 µL aliquots were collected in duplicate, and the enzymatic reaction was immediately stopped by adding 2 mL of absolute ethanol. Glucose content in the solution was quantified colorimetrically using a glucose oxidase kit (GODPOD 4058, Giesse Diagnostic snc, Rome, Italy) at 510 nm. Each sample was analyzed in technical duplicate. A conversion factor of 0.9 was used to calculate the percentage of digested starch at each time interval, converting from mono- to polysaccharides. The hydrolysis index (HI) was calculated as the ratio between the area under the curve (0–180 min) for each sample and the corresponding area obtained from the in vitro digestion of white bread, expressed as a percentage over the same period. Finally, the predicted glycemic index (pGI) was calculated using the formula: pGI = 8.198 + 0.862 × HI [45].

### 2.10. Antioxidant Activities Assays

The total antioxidant capacity of pigmented wheat flours and bran-enriched fractions was measured using the ferric reducing antioxidant power (FRAP) assay and the 2, 2′-azino-bis (3-ethylbenzothiazoline-6-sulfonic acid) (ABTS^•+^) radical scavenging assay as follows. Pasta was cooked as described in the previous paragraph. All extractions were performed on a single homogenized sample per genotype and fraction, with subsequent analytical measurements carried out as technical replicates. Cooked pasta and flour were frozen at −80 °C and, after lyophilization, cooked pasta was ground into a fine powder using a grain coffee mill. Total phenolic compounds were extracted from 1 g of powdered sample by mixing it with 15 mL of methanol–water (80:20, *v*/*v*). The mixture was shaken overnight at room temperature in an orbital shaker at 400 rpm. The extract was then centrifuged at 4000 rpm for 5 min, and the supernatant was collected and stored at 4 °C. The extraction procedure was repeated twice more, and the combined extracts were finally filtered through a 0.45 µm Millipore membrane filter.

#### 2.10.1. Ferric-Reducing Antioxidant Power (FRAP) Assay

FRAP assays were performed following the methodology reported in [46]. Briefly, the FRAP reagent was freshly prepared daily by combining 300 mmol/L sodium acetate buffer (pH 3.6), 10 mmol/L TPTZ dissolved in 40 mmol/L HCl, and 20 mmol/L ferric chloride hexahydrate (FeCl_3_·6H_2_O) solution in a 10:1:1 ratio. Before use, the reagent was incubated in a water bath at 37 °C for 10 min. A total of 10 µL of sample/standard/blank was mixed with 160 µL of the FRAP reagent. The solution was incubated at 37 °C directly in the Thermo Scientific™ Multiskan™ GO Microplate Spectrophotometer (hermo Fisher Scientific Inc., Waltham, MA, USA) and, after 30 min, the absorbance was recorded at 593 nm. A standard calibration curve ranging from 15 µM to 1000 µM was established using a ferrous sulfate (FeSO_4_) solution, and the antioxidant capacity was expressed as µmol FeSO_4_ L^−1^ g^−1^ dry weight of the sample.

#### 2.10.2. Trolox Equivalent Antioxidant Capacity (TEAC) Assay

The ABTS^•+^ radical scavenging activity was determined using the OxiSelect™ Trolox Equivalent Antioxidant Capacity (TEAC) Assay Kit (ABTS, Cell Biolabs INC., San Diego, California, USA) following the manufacturer’s protocol. Briefly, a total of 150 μL of ABTS was added to 25 μL of sample/Trolox standard and thoroughly mixed. The reaction mixture was incubated at room temperature for 5 min. Absorbance measurements were taken at 405 nm using an automated microplate reader (Thermo Scientific™ Multiskan™ GO Microplate Spectrophotometer). A Trolox standard curve was generated, and the results were expressed as μmol of Trolox equivalents (TE) per gram of dry weight (DW).

Both analyses, FRAP and Trolox, were performed in triplicate (three technical replicates for each sample), and results were expressed as mean ± standard deviation (SD). Data were analyzed using one-way ANOVA followed by Tukey’s post hoc test to determine statistical significance (*p* < 0.05).

## 3. Results

### 3.1. Physicochemical Properties and Fiber Content of Grain

**Figure 1:**
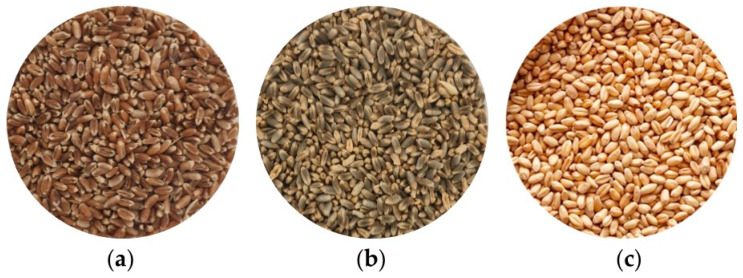
Bread wheat grain. (**a**) Purple variety Vanilnoir; (**b**) Blue variety Purendo; (**c**) Peralba.

Vanilnoir (purple grain) and Purendo (blue grain) were selected for the qualitative assessment of pigmented wheat (Figure 1a,b). The non-pigmented cultivar Peralba was used as control (Figure 1c). Grain quality was evaluated using near-infrared transmittance (NIT) spectroscopy and through biochemical analyses performed on wholemeal flour (Table S2). The physicochemical analysis showed similar moisture levels among the three genotypes (11.3–11.6%). Purendo and Vanilnoir exhibited the highest protein content (≈16%), while Peralba showed a lower value (11.2%). For test weight, Peralba recorded the highest value (84.9%), whereas Vanilnoir presented the lowest (79.8%).

Kernel hardness was assessed using the Single Kernel Characterization System. Vanilnoir showed the highest hardness index (78), classifying it as a hard wheat. Purendo displayed the lowest value (42), corresponding to a soft wheat type, while Peralba (68.7) fell within the medium-hardness category (Table S2). These results were supported by sequence analysis of the *PinA* and *PinB* genes: Purendo carries the wild-type *PinA-D1a* and *PinB-D1a* alleles, consistent with a soft texture, whereas Vanilnoir carries the *PinB-D1b* allele, associated with hard texture. The SDS sedimentation test indicated stronger gluten in Vanilnoir (12.55 cm) and Purendo (11.6 cm) compared with Peralba (9.05 cm) (Table S2). SDS-PAGE analysis of glutenin fractions revealed distinct high-molecular-weight glutenin subunit (HMW-GS) compositions: Vanilnoir showed subunits 7, 2 + 12; Purendo showed 1, 6 + 8, 5 + 10; and Peralba presented 7 + 8, 2 + 12. These differences highlight the heterogeneity of gluten protein profiles among pigmented wheat varieties and may influence their technological properties. No significant differences were observed in fiber content among the genotypes (Table S3). Vanilnoir had the highest percentage of water-extractable arabinoxylans (5.67%) but the lowest total arabinoxylan content (46.76%), whereas Peralba showed the highest total arabinoxylan value (53.38%). Amylose and β-glucan contents were relatively consistent across genotypes, ranging from 30.57% to 32.30% and 0.40% to 0.52%, respectively (Table S3).

### 3.2. Composition and Rheological Parameters

The grain samples were divided into two portions: one was processed using a traditional milling system to obtain flour, and the other was processed using an innovative method involving micronization followed by air-classification to produce semi-wholemeal flours. The products from the innovative method were then subjected to air-classification at an airflow rate of 250 m^3^/h, yielding two fractions: a fine fraction (F250) and a coarse fraction (G250). Analyses performed on the traditional flour and the micronized/air-classified products (F250 and G250) included compositional and rheological parameters (Table 1).

Ash content determination revealed a higher percentage in the products obtained via micronization and air-classification compared to the respective traditional flours. Specifically, the F250 samples showed an increase ranging from a minimum of over 150% to a maximum exceeding 180%, with the highest value observed in the Purendo variety. In the G250 samples, the increase ranged from a minimum of over 80% to a maximum exceeding 240%, again with the highest value recorded for Purendo (Table 1).

Gluten content was assessed both quantitatively and qualitatively. The results confirmed that the two pigmented varieties, Vanilnoir and Purendo, exhibited high protein content and gluten values (exceeding 13% in Vanilnoir and 11% in Purendo) in both the traditional flours and the air-classified products. However, gluten quality was found to be relatively low, with a further decline observed in the F250 samples (Table 1).

Falling Number results in the range of 362–559 s indicate a very low α-amylase activity across all samples, with the lowest value recorded in Peralba F250 fraction (362 s). This suggests limited starch degradation, a desirable trait for pasta production, as high enzymatic activity can compromise dough structure and final product quality by promoting excessive starch hydrolysis during processing. A slight decrease in Falling Number values was observed in the F250 fractions of all genotypes, likely due to the redistribution or partial removal of enzymatically active outer grain layers during air classification. For instance, in Purendo fractions, Falling Number decreased from 434 s in flour to 362 s in F250; similarly, Vanilnoir showed an improvement from 559 s to 507 s in the F250 fraction.

Therefore, although all samples show very low α-amylase activity, the consistent decrease in the F250 fraction is a direct result of the altered enzyme distribution caused by the milling and fractionation process (Table 1).

The alveographic values of the traditional flours were generally low, a characteristic attributed to their high extensibility. In contrast, the F250 fractions, due to their higher bran content, exhibited a marked reduction in extensibility (L value) and a corresponding increase in dough tenacity (*p* value). This shift in rheological properties is clearly demonstrated by the P/L ratio. The ratios for the traditional flours, initially ranging between 0.20 and 0.30, increased significantly after air-classification, reaching values between 2.7 and 4.14, representing a drastic alteration in dough rheology (Table 1).

Farinograph analyses revealed that the air-classified F250 fractions consistently exhibited higher water absorption than the corresponding traditional flours, with an average increase of approximately 22%. This enhancement is directly attributed to the higher concentration of bran and fibrous components in the fine fraction, which possess greater water-binding capacities [47]. Among the genotypes, the Vanilnoir F250 fraction showed the highest water absorption (67.3%), followed by Purendo F250 (62.4%) and Peralba F250 (60.3%), reflecting their distinct fiber and protein compositions.

However, the dough softening degree (ICC/12 min after peak), a key indicator of dough stability, deteriorated significantly in all F250 samples. The extent of the decline varied by genotype.

Vanilnoir F250 experienced the most pronounced weakening, with a 38% reduction in stability compared to its flour (from 73 UF to 45 UF), Purendo F250 showed a 22% decrease (from 64 to 50 UF), and Peralba F250 was the least affected, with only a 7% reduction (from 69 to 64 UF). These results suggest that Vanilnoir, despite its higher protein content, is more sensitive to the negative effects of bran-rich fractions on dough stability, probably due to the nature of its gluten network or higher fiber content interfering with gluten development. Peralba, with lower protein and more balanced fiber distribution, maintained greater dough consistency even after air classification. Both traditionally milled (flours) and turbo-separated samples (F250 and G250) were also evaluated for yellow color intensity and browning index (Table S4). Regarding yellow color, F250 samples showed lower values compared to flours, ranging from a minimum reduction of −7% in Peralba to −25% in Vanilnoir. In contrast, G250 samples exhibited an increase in yellow color, with a minimum rise of 19% in Vanilnoir and a maximum increase of 50% in the other two varieties. The browning index increased significantly in both fractions (F250 and G250), with an average growth of over 60% in F250 samples, ranging from a minimum of 120% in Vanilnoir to a maximum exceeding 200% in the other two samples (Table S4).

### 3.3. Evaluation of Pasta-Making Performance and Quality Characteristics of Pigmented Wheat Flour Fractions

**Figure 2:**
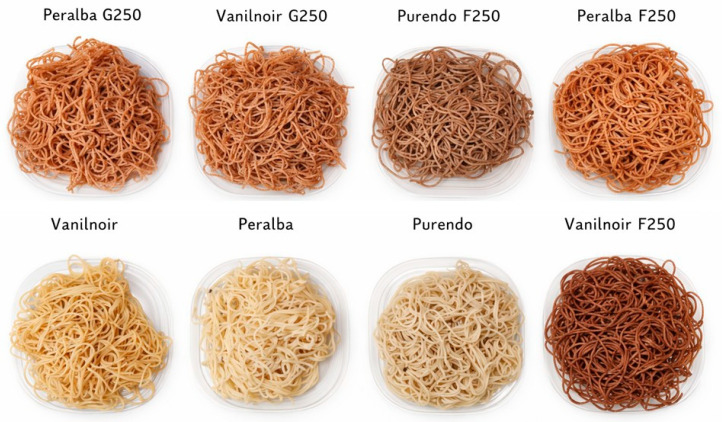
Cooked pasta produced from different genotypes using different milling methods.

Pasta-making trials were conducted using the F250 and G250 milling fractions, applying a drying profile at 50 °C to enhance pasta quality while preserving the intrinsic properties of each genotype retained through the milling process. Pasta produced from the whole flour of each genotype it was used as a control (Figure 2).

Among the control samples, pasta made from Peralba flour exhibited the longest cooking time (10′30″), but also the lowest values for stickiness (10), firmness (40), and bulkiness (30), resulting in noticeable textural weakness and a marked loss of structure during tasting (Table 2).

The use of milling fractions (F250 and G250) generally led to a reduction in cooking time across all genotypes, except for Purendo G250, which showed a longer cooking time than both the flour and F250-based samples. A decrease in cooked pasta weight was observed for both genotypes when using the milling fractions, likely due to altered hydration and swelling properties (Table 2).

For the Purendo genotype, the use of air-classified milling fractions significantly increased cooked pasta stickiness and bulkiness compared to the traditional flour-based pasta, negatively impacting overall quality. Stickiness values rose from 40 (control) to 80 (F250) and 70 (G250). Similarly, bulkiness increased from 50 (control) to 80 (F250) and 60 (G250).

Although firmness improved with both fractions, reaching 65 in F250 and G250 compared to 50 in the control, this beneficial effect was offset by the excessive increase in stickiness and bulkiness, particularly in the F250 fraction. These results indicate that, for the Purendo genotype, while milling fractions can enhance pasta firmness, they concurrently compromise its overall technological quality due to undesirable increases in surface adhesiveness and volume expansion.

In contrast, the Vanilnoir genotype showed a more favorable response to the G250 fraction. While firmness decreased slightly from 70 in the control pasta to 60 in the F250 and 50 in the G250 fraction, the G250 formulation significantly reduced stickiness (55) and bulkiness (50) compared to both the traditional flour-based pasta (65 for both traits) and the F250 pasta (80 for both traits). This indicates that, despite a modest reduction in firmness, the G250 fraction improved the overall textural quality of Vanilnoir pasta by effectively minimizing its two most undesirable attributes: stickiness and bulkiness.

Regarding nutritional composition (Table 3), the most statistically significant differences among the genotypes were observed in spaghetti made from traditional flours, particularly between Vanilnoir and the other two varieties. Spaghetti from Vanilnoir exhibited significantly lower moisture and higher ash contents than those from Peralba and Purendo. Furthermore, Peralba had a considerably lower protein content than both Purendo and Vanilnoir. No significant inter-genotypic differences were found in total starch content for the flour-based pasta. The use of the F250 fraction induced consistent compositional changes across all three genotypes compared to their respective traditional flours. Specifically, the F250 spaghetti showed marked increases in ash, lipid, and protein content, alongside a significant reduction in total starch (Table 3). Ash content increased significantly in the F250 fraction for all genotypes. For instance, in Peralba, it more than doubled from 0.66% to 1.42%, while strong increases were also seen in Purendo (from 0.64% to 1.86%) and Vanilnoir (from 0.74% to 1.84%). A consistent rise in protein content was also observed. In Purendo, protein increased from 13.03% to 15.62%, and in Vanilnoir, it increased from 14.88% to 16.49%. Peralba showed a more modest increase from 9.22% to 10.76%. Lipid content followed the same trend, with F250 fractions showing higher values. The most substantial increase was observed in Purendo, where lipid content rose from 0.19% to 0.51%. In contrast, total starch content decreased significantly in the F250 fractions across all genotypes: Peralba showed a decline from 69.16% to 59.13%, Purendo showed a decrease from 70.16% to 54.83%, and Vanilnoir showed a decrease from 61.79% to 52.45%. These results suggest that the air-classification process producing the F250 fraction enriches components associated with the grain’s outer layers, namely minerals, proteins, and lipids, while depleting the starchy endosperm fraction (Table 3).

**Figure 3:**
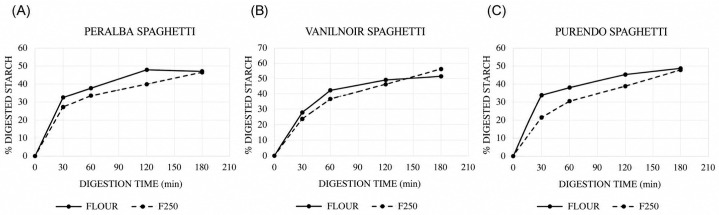
Starch in vitro digestion kinetics of Peralba (**A**), Purendo (**B**) and Vanilnoir (**C**) spaghetti over time. The percentage of digested starch was measured at different time points (0–180 min) for spaghetti produced from flour (solid line) and F250 (dashed line). Data points represent mean values.

The Hydrolysis Index (HI) and predicted Glycemic Index (pGI) did not show statistically significant differences among the various types of spaghetti made from the flours of different genotypes. However, spaghetti produced from the F250 fraction exhibited a lower HI and pGI across all genotypes, except for Vanilnoir (Table 4, Figure 3).

### 3.4. Antioxidant Capacity of Pigmented Wheat Flours and Enriched Fractions: FRAP and TEAC Assays

The antioxidant capacity of pigmented wheat flours and their bran-enriched fractions was assessed using both the Ferric Reducing Antioxidant Power (FRAP) and Trolox Equivalent Antioxidant Capacity (TEAC) assays, offering a comprehensive evaluation of how milling and cooking affect the bioactive potential of these wheat-derived products. Antioxidant activity varied significantly across wheat varieties and milling methods, as well as in their respective pasta products. Among the varieties tested, the highest FRAP values for both flour and pasta were observed in the blue wheat cultivar Purendo, particularly in the F250 and G250 fractions. In contrast, Peralba white flour exhibited the lowest FRAP activity, with values of 0.103 mmol FeSO_4_/g DW in flour and 0.072 mmol FeSO_4_/g DW in its corresponding pasta. In terms of TEAC, Vanilnoir consistently showed the highest ABTS^•+^ scavenging activity, especially in both white flour and F250 fractions. Notably, pasta produced from Vanilnoir F250 retained a high TEAC value (0.490 mmol TE/g DW), further underscoring the strong antioxidant profile of this genotype. Both assays confirmed that milling significantly influences the antioxidant properties of flour (Table 5). In the FRAP assay, flours derived from whole grain fractions (F250 and G250) demonstrated superior iron-reducing capacity compared to white flour. Purendo F250 displayed the highest FRAP value (~0.36 mmol FeSO_4_/g DW), followed by Vanilnoir F250 (~0.32 mmol FeSO_4_/g DW) and Peralba F250 (~0.23 mmol FeSO_4_/g DW). A similar pattern emerged in the TEAC assay (Table 5), where Vanilnoir flour consistently recorded the highest values (0.596 mmol TE/g DW in white flour and 0.584 mmol TE/g DW in F250). Conversely, Purendo exhibited a marked decline in antioxidant activity from white flour (0.595 mmol TE/g DW) to the F250 (0.510 mmol TE/g DW) and G250 (0.422 mmol TE/g DW) fractions. Peralba followed a comparable trend, with TEAC values decreasing from 0.462 mmol TE/g DW in white flour to 0.370 mmol TE/g DW in G250. These results suggest that while bran enrichment through F250 milling enhances antioxidant potential, the overall effect of milling is complex, likely governed by how the process influences the retention of bioactive compounds. Cooking further modulated antioxidant activity, as revealed by both FRAP and TEAC analyses (Table 5). The FRAP assay indicated a cultivar- and fraction-dependent response to thermal processing. In particular, Vanilnoir F250 and Peralba white flour and F250 experienced a notable reduction in FRAP values following cooking, while Purendo-derived samples maintained much of their antioxidant potential. These patterns were corroborated by the TEAC assay, which highlighted significant losses in radical scavenging activity after cooking, especially in pastas derived from white flour. For Vanilnoir, TEAC values dropped from 0.596 mmol TE/g DW in white flour to 0.188 mmol TE/g DW in the corresponding pasta, with a less pronounced, though still significant, reduction in the F250-derived product (from 0.584 to 0.490 mmol TE/g DW). Purendo exhibited a similar trend, with TEAC decreasing sharply in white flour pasta (0.230 mmol TE/g DW vs. 0.595 mmol TE/g DW in flour), while F250 and G250-derived pastas retained higher antioxidant levels (0.420 and 0.416 mmol TE/g DW, respectively). In Peralba, the most substantial antioxidant loss occurred in white flour pasta (0.264 mmol TE/g DW vs. 0.462 mmol TE/g DW in flour), although pastas made from F250 and G250 showed comparatively better antioxidant preservation (0.357 and 0.273 mmol TE/g DW, respectively).

## 4. Discussion

Wheat is a cornerstone of the global food supply, providing a significant proportion of daily caloric intake and forming the basis of numerous staple foods [48]. Despite its central role, conventional wheat products often lack essential micronutrients and bioactive compounds beneficial for human health. Enhancing the nutritional value of wheat through genetic biofortification and innovative processing technologies represents a strategic pathway to address both dietary deficiencies and consumer demand for functional foods [2,20]. This study demonstrates the feasibility and potential of combining anthocyanin-rich pigmented wheat genotypes with advanced milling technologies, micronization and air-classification to produce nutritionally enriched fractions with favorable technological properties. By evaluating two colored wheat varieties, Purendo (blue) and Vanilnoir (purple), in comparison with a conventional non-pigmented control (Peralba), we highlight how genotype and milling approach co-determine the nutritional quality, antioxidant capacity, and end-use performance of wheat-derived foods.

In agreement with prior studies [49,50], both colored genotypes exhibited significantly higher protein contents (>16%) than the white control, which is particularly advantageous in pasta production, where protein quantity and gluten strength are key quality determinants [51]. Vanilnoir and Purendo also displayed distinct glutenin subunit compositions, revealed by sedimentation tests and SDS-PAGE analyses, which align with known effects of gluten polymorphisms on dough viscoelasticity and pasta firmness [52,53]. Specifically, Vanilnoir was characterized by the HMW-GS combination 7, 2 + 12, typically associated with weaker gluten strength and reduced dough elasticity. In contrast, Purendo carried the subunits 1, 6 + 8, and 5 + 10, which are linked to stronger gluten networks, improved dough viscoelasticity, and superior pasta-making quality [52]. Additionally, differences in grain texture were confirmed by puroindoline allele analysis: Vanilnoir’s hard texture was associated with the *PinB-D1b* allele, while Purendo maintained a soft endosperm due to the presence of wild-type *PinA-D1a* and *PinB-D1a* [54], with implications for milling performance and starch damage. Air-classification proved effective in concentrating proteins, ash, and anthocyanins in F250 and G250 fractions, consistent with literature describing micronutrient and fiber accumulation in outer kernel layers [21,55,56]. The F250 fractions of pigmented genotypes exhibited the highest FRAP and TEAC values, with Purendo and Vanilnoir reaching up to 0.36 mmol FeSO_4_/g DW and 0.596 mmol TE/g DW, respectively, underscoring the nutritional advantage of targeting anthocyanin-rich tissues. These findings align with reports of elevated phenolic and flavonoid concentrations in pigmented wheat, which contribute significantly to antioxidant capacity [57]. Despite nutritional benefits, enrichment adversely affected dough rheology. Bran-rich F250 fractions exhibited reduced extensibility and elevated P/L ratios, reflecting bran-induced disruption of gluten network formation, as reported for wholegrain systems [58,59].

This effect was particularly pronounced in Vanilnoir, where increased water competition from bran fibers contributed to dough softening [60]. In general, the incorporation of fiber into pasta formulations is known to reduce firmness due to the physical disruption of the gluten network by insoluble fibrous particles [56]. Pasta trials confirmed these effects: although Purendo-based F250 pasta exhibited enhanced firmness, it also showed undesirable stickiness and bulkiness, consistent with previous findings on wholegrain pasta systems [59]. Conversely, Vanilnoir G250 pasta maintained acceptable sensory characteristics, suggesting a more favorable balance between nutritional enrichment and technological performance. However, this sensory evaluation relied on a relatively small panel of trained experts. Future research involving larger, more diverse consumer cohorts will be required to validate these findings.

Beyond processing behavior, the study also explored antioxidant stability during thermal treatment and among milling method. These results provide compelling evidence for the role of both genotype and processing methods, particularly milling and cooking, in shaping the antioxidant properties of pigmented wheat-derived products. The observed variation among genotypes reinforces the importance of genetic background in determining the accumulation and retention of bioactive compounds, especially phenolic acids, which are primarily located in the bran and aleurone layers. In this context, the consistently higher antioxidant activity observed in Vanilnoir and Purendo suggests that pigmented wheats, due to their distinct polyphenolic profiles, are inherently superior to white wheat (Peralba) in terms of their health-promoting potential.

The milling process emerged as a critical factor influencing antioxidant capacity. Notably, the bran-enriched F250 and G250 fractions exhibited enhanced FRAP and TEAC values compared to white flour, consistent with the concentration of antioxidant compounds in the outer layers of the grain. However, the results also reveal that this enrichment is not uniformly effective across genotypes. For instance, while Vanilnoir and Peralba showed marked improvements in antioxidant capacity upon fractionation, Purendo displayed a decrease in TEAC values from white flour to F250 and G250 fractions, an unexpected trend that may reflect the predominant accumulation of anthocyanins in the aleurone layer, where phenolic compounds are less extractable or more tightly bound to the cell wall matrix, potentially limiting their recovery and interfering with antioxidant assays. Cooking had a significant and often detrimental impact on antioxidant activity, especially in white flour-derived pasta. The thermal degradation of phenolic compounds and possible structural changes in the food matrix likely contribute to this loss. Nevertheless, bran-enriched pasta, particularly from F250 and G250 fractions, demonstrated a greater ability to retain antioxidant activity post-cooking. This supports the hypothesis that the presence of higher levels of phenolics and associated compounds in these fractions confers greater thermal stability or protection against oxidative degradation during processing. The differential responses to cooking among genotypes further highlight the complexity of the interaction between genotype, milling, and thermal treatment.

Despite its high protein content, Vanilnoir exhibited the poorest dough stability when bran-rich fractions were used. This suggests that protein quantity alone is not a reliable predictor of technological performance; rather, the specific composition of gluten subunits and their interaction with fiber play a crucial role.

Interestingly, G250 fractions, although less fiber-enriched than F250, achieved a better balance between processing behavior and nutritional enhancement in Vanilnoir. For example, G250 pasta from this genotype showed lower stickiness and bulkiness while maintaining good antioxidant retention, indicating greater suitability for pasta applications.

Pasta made from F250 fractions of all genotypes showed significantly lower predicted glycemic index (pGI) values compared to those made from conventional flour.

This effect can be primarily attributed to the higher dietary fiber content and the consequent reduction in starch digestibility. Dietary fiber is known to limit the accessibility of digestive enzymes to starch granules, thereby slowing the rate of starch hydrolysis and glucose release during in vitro digestion. As a result, fiber-enriched cereal products generally show a reduced glycemic response compared to refined counterparts [61]. In pasta systems specifically, the incorporation of dietary fibers has been reported to reduce the predicted glycemic index by up to 40%, mainly due to structural modifications in the pasta matrix that hinder starch degradation during digestion [62]. In addition, the presence of fiber and other non-starch polysaccharides can modify the microstructure of the pasta matrix, increasing viscosity in the digestive environment and creating physical barriers that limit enzyme diffusion and starch accessibility. These mechanisms reduce the interaction between amylolytic enzymes and starch, ultimately slowing glucose release and lowering the release of glucose during the in vitro digestion [63]. However, pGI values derived from in vitro assays may not fully reflect in vivo glycemic responses, and further clinical validation would be required.

While laboratory-scale tests provided valuable insights, future studies should include larger-scale sensory evaluations, including consumer-based panels, and pilot-scale production to assess consumer acceptance and industrial feasibility. Overall, the results highlight the importance of genotype-specific milling strategies for developing functional pasta products with enhanced nutritional profiles and satisfactory technological performance.

In particular, F250 milling offers a promising approach for concentrating health-promoting compounds while reducing starch content. Among the genotypes tested, Vanilnoir stands out for its compositional strength and favorable processing characteristics, making it especially suitable for functional pasta production.

## 5. Conclusions

This study highlights the potential of combining anthocyanin-rich wheat genotypes with advanced milling technologies, specifically micronization and air classification, to develop functional flour fractions enriched in antioxidant compounds. Both Purendo and Vanilnoir benefited nutritionally from bran enrichment; however, only Vanilnoir retained favorable technological and sensory characteristics compatible with pasta production. Among the milling strategies, the F250 process was particularly effective in concentrating bioactive compounds and preserving antioxidant capacity after cooking, although its effectiveness varied by genotype. These findings support the adoption of genotype-specific processing strategies aimed at enhancing the health-promoting qualities of wheat-based foods without compromising their technological performance. Future research should focus on evaluating anthocyanin bioaccessibility, digestive stability, and consumer acceptance under industrially relevant conditions to facilitate the commercial uptake of pigmented wheat-based functional products. This aligns with increasing evidence that whole grain consumption and minimal processing are essential to maximizing the nutritional value of cereal foods. Moreover, it underscores the importance of selecting appropriate genotype–processing combinations as a strategy to improve the nutritional profile of staple products such as pasta. Further investigations are warranted to characterize the phenolic composition of enriched fractions in greater detail and to assess the bioavailability and in vivo functionality of their antioxidant constituents, thereby informing breeding programs and food innovation efforts.

## Figures and Tables

**Table 1:** Composition and rheological parameters of F250 and G250 air-classified fractions from micronized whole grain, compared with control flour and micronized samples.

Genotype	Peralba				Purendo				Vanilnoir			
Formulations	Flour	Micronized	F250	G250	Flour	Micronized	F250	G250	Flour	Micronized	F250	G250
Protein (%)	9.72 ± 0.07 ^g^	10.69 ± 0.07 ^f^	10.73 ± 0.06 ^f^	10.43 ± 0 ^f^	15.12 ± 0.04 ^e^	17.43 ± 0.01 ^c^	17.38 ± 0.04 ^c^	19.82 ± 0.14 ^a^	16.23 ± 0.01 ^d^	18.11 ± 0.01 ^b^	18.55 ± 0.05 ^b^	16.48 ± 0.37 ^d^
Ash %	0.63 ± 0 ^1^	1.59 ± 0.01 ^e^	1.64 ± 0.01 ^e^	1.46 ± 0.01 ^f^	0.72 ± 0 ^h^	1.94 ± 0.01 ^c^	2.08 ± 0.01 ^b^	2.50 ± 0 ^a^	0.81 ± 0.01 ^g^	1.86 ± 0 ^d^	2.08 ± 0.01 ^b^	1.52 ± 0.01 ^f^
Gluten % s.s.	7.25 ± 0.06 ^d^		6.59 ± 0.06 ^c^		11.72 ± 0.03 ^b^		11.51 ± 0.09 ^b^		13.22 ± 0.14 ^a^		2.08 ± 0.17 ^e^	
Gluten Index	96 ± 0.48 ^a^		98 ± 1.55 ^a^		85 ± 0.55 ^b^		65 ± 1.92 ^c^		68 ± 0.5 ^c^		56 ± 1.84 ^d^	
Falling number	409 ± 1.41 ^c^		362 ± 2.12 ^d^		434 ± 4.95 ^c^		401 ± 2.83 ^cd^		559 ± 19.80 ^a^		507 ± 16.97 ^b^	
**Alveograph parameters**												
P	43 ± 4.15 ^c^		120 ± 6.02 ^a^		29 ± 1.52 ^d^		99 ± 7.53 ^b^		46 ± 1.71 ^c^		100 ± 2.88 ^b^	
L	155 ± 18.61 ^c^		29 ± 2.3 ^a^		156 ± 41.64 ^c^		31 ± 2.45 ^a^		207 ± 6.61 ^b^		37 ± 4.87 ^a^	
W	176 ± 11.91 ^a^		147 ± 4.6 ^bc^		94 ± 15.84 ^d^		125 ± 6.07 ^c^		176 ± 3.5 ^a^		141 ± 8.76 ^c^	
P/L	0.28 ± 0.06 ^c^		4.14 ± 0.46 ^a^		0.19 ± 0.05 ^c^		3.19 ± 0.46 ^b^		0.22 ± 0.01 ^b^		2.7 ± 0.39 ^c^	
**Farinograph parameters**												
Consistency (UF)	496		506		481		514		492		509	
WA (%)	54.8		64.2		54.8		66.4		61.4		71.2	
WA 500UF (%)	54.7		64.3		54.3		66.8		61.2		71.4	
WA 14%	52.9		60.3		51.9		62.4		58.9		67.3	
DDT (MIN.)	2.7		4.3		4		4.8		4.8		4.7	
DS (MIN.)	7.5		4.8		4		3.7		5		3.5	
Softening Degree 10 UF	48		42		56		49		38		41	
Softening Degree 12 UF	69		64		88		70		73		45	
Farinograph QN	78		76		68		76		93		81	

P = tenacity; L = extensibility; P/L = curve configuration ratio; W = deformation energy; WA = water absorption; DDT = dough developing time; DS = Dough stability. Values are reported as mean ± standard deviation. A One-way ANOVA analysis was performed for each row, followed by Tukey’s multiple comparisons post hoc test. Values that do not share the same letter are statistically different (*p* < 0.01). Softening Degree 10 UF = (10 Min After Start) UF; Softening Degree 12 UF = (Icc/12 Min After Peak) UF; Farinograph Quality Number = Farinograph QN.

**Table 2:** Cooking time and quality traits of pasta.

Genotype	Peralba	Purendo	Vanilnoir	Peralba	Purendo	Vanilnoir	Peralba	Purendo	Vanilnoir
Formulation	Flour			F250			G250		
Cooking time	10.30	9.15	8.40	9.30	9.00	8.30	8.30	9.5	8.2
Diameter	1.66 ± 0.05	1.62 ± 0.03	1.67 ± 0.05	1.7 ± 0.04	1.68 ± 0.03	1.72 ± 0.05	1.64 ± 0.08	1.75 ± 0.06	1.57 ± 0.04
Weight	251.33	250.65	231.14	250.41	237.17	228.47	244.96	237.32	240.69
Stickiness	10 ± 0 ^e^	40 ± 0 ^d^	65 ± 7 ^bc^	60 ± 0 ^bc^	80 ± 0 ^a^	80 ± 0 ^a^	50 ± 0 ^cd^	70 ± 0 ^ab^	55 ± 7 ^c^
Firmness	40 ± 0 ^c^	50 ± 0 ^c^	70 ± 0 ^a^	50 ± 0 ^c^	65 ± 7.07 ^ab^	60 ± 0 ^abc^	40 ± 0 ^c^	65 ± 7.07 ^a^	50 ± 0 ^c^
Bulkiness	30 ± 0 ^d^	50 ± 0 ^c^	65 ± 7 ^b^	60 ± 0 ^b^	80 ± 0 ^a^	80 ± 0 ^a^	50 ± 0 ^c^	60 ± 0 ^b^	50 ± 0 ^c^

A One-way ANOVA analysis was performed for each row, followed by Tukey’s multiple comparisons post hoc test. Values that do not share the same letter are statistically different (*p* < 0.05).

**Table 3:** Chemical composition of cooked Peralba, Purendo, and Vanilnoir spaghetti obtained from flour and fine fraction (F250). % Moisture indicates the moisture content of the sample.

	Peralba		Purendo		Vanilnoir	
	Flour	F250	Flour	F250	Flour	F250
% Moisture	10.96 ± 0.02 ^a^	10.92 ± 0.25 ^a^	11.08 ± 0.01 ^a^	10.56 ± 0.07 ^b^	10.62 ± 0.09 ^b^	10.45 ± 0.11 ^b^
% Ash	0.66 ± 0.01 ^d^	1.42 ± 0.03 ^b^	0.64 ± 0.00 ^d^	1.86 ± 0.01 ^a^	0.74 ± 0.00 ^c^	1.84 ± 0.01 ^a^
% Proteins	9.22 ± 0.31 ^c^	10.76 ± 0.55 ^c^	13.03 ± 0.49 ^b^	15.62 ± 0.18 ^a^	14.88 ± 0.09 ^a^	16.49 ± 0.02 ^a^
% Lipids	0.55 ± 0.19 ^a^	0.59 ± 0.13 ^a^	0.19 ± 0.06 ^b^	0.51 ± 0.03 ^a^	0.26 ± 0.04 ^a^	0.34 ± 0.07 ^a^
% Total starch	69.16 ± 1.69 ^a^	59.13 ± 0.23 ^b^	70.16 ± 0.32 ^a^	54.83 ± 0.96 ^c^	61.79 ± 4.08 ^b^	52.45 ± 1.12 ^c^

A One-way ANOVA analysis was performed for each row, followed by Tukey’s multiple comparisons post hoc test. Values that do not share the same letter are statistically different (*p* < 0.05).

**Table 4:** Calculated hydrolysis index and predicted glycemic index (pGI) of Peralba, Purendo, and Vanilnoir spaghetti made from flour and fine fraction (F250).

Genotype	HI		pGI	
	Flour	F250	Flour	F250
Peralba	62.55 ± 0.37 ^a^	54.93 ± 0.9 ^b^	62.11 ± 0.32 ^a^	55.54 ± 0.78 ^b^
Purendo	66.42 ± 1.33 ^a^	55.23 ± 0.88 ^b^	65.45 ± 1.14 ^a^	55.80 ± 0.76 ^b^
Vanilnoir	65.92 ± 0.07 ^a^	63.11 ± 1.22 ^a^	65.02 ± 0.06 ^a^	62.60 ± 1.06 ^a^

An unpaired *t*-test was performed between spaghetti obtained from flour and those obtained from F250 within the same genotype. Values that do not share the same letter are statistically different (*p* < 0.05).

**Table 5:** Comparison of antioxidant properties of flour and cooked pasta for each variety and milling method.

Variety	Milling Method	Food Type	FRAP (mmol FeSO_4_/g DW)	TEAC (mmol TE/g DW)
Vanilnoir	Flour	Flour	0.124 ± 0.002 ^ef^	0.596 ± 0.004 ^a^
		Pasta	0.112 ± 0.021 ^fg^	0.188 ± 0.003 ^e^
	F250	Flour	0.316 ± 0.011 ^ab^	0.584 ± 0.025 ^a^
		Pasta	0.278 ± 0.010 ^bc^	0.490 ± 0.017 ^b^
Purendo	Flour	Flour	0.115 ± 0.006 ^fg^	0.595 ± 0.006 ^a^
		Pasta	0.094 ± 0.009 ^g^	0.230 ± 0.016 ^d^
	F250	Flour	0.362 ± 0.032 ^a^	0.510 ± 0.007 ^b^
		Pasta	0.317 ± 0.011 ^ab^	0.420 ± 0.012 ^c^
	G250	Flour	0.357 ± 0.022 ^a^	0.422 ± 0.017 ^c^
		Pasta	0.363 ± 0.015 ^a^	0.416 ± 0.025 ^c^
Peralba	Flour	Flour	0.103 ± 0.006 ^fg^	0.462 ± 0.003 ^b^
		Pasta	0.072 ± 0.004 ^g^	0.264 ± 0.008 ^d^
	F250	Flour	0.232 ± 0.015 ^cd^	0.419 ± 0.015 ^c^
		Pasta	0.191 ± 0.006 ^de^	0.357 ± 0.010 ^c^
	G250	Flour	0.207 ± 0.001 ^de^	0.370 ± 0.007 ^c^
		Pasta	0.200 ± 0.006 ^de^	0.273 ± 0.024 ^d^

A One-way ANOVA analysis was performed for each column, followed by Tukey’s multiple comparisons post hoc test. Values followed by a different letter are significantly different (*p* < 0.05).

## Data Availability

The original contributions presented in this study are included in the article/Supplementary Materials. Further inquiries can be directed to the corresponding authors.

## Supplementary Materials

The following supporting information can be downloaded at https://www.mdpi.com/article/10.3390/foods15091598/s1, Table S1: Genotypes characterized in this study; Table S2: Physicochemical and Rheological Properties of whole flour; Table S3: Arabinoxylan, amylose, and β-glucan contents; Table S4: Evaluation of Color Attributes and Browning Index in Wheat Flours and Fractions.
