# Supplementary material for: Bran-Enriched Fractions from Blue and Purple Wheat Improve Antioxidant Potential and Nutritional Profile
Source: Foods. 2026 May 5;15(9):1598. doi: 10.3390/foods15091598 (PMC13164409; doi:10.3390/foods15091598)
Supplement: Supplementary file 1 [file foods-15-01598-s001.zip › foods-4282009-supplementary.pdf]

Supplemental material

Table S1. Genotype characterized in this study.

Genotype	Breeder/ Pedigree	Coloration of the grains
Vanilnoir	Agroscope/DSP (CH).<br>RUNAL/W'ST479-77//KONINI	Purple
Purendo	Crop Development Center, Saskatoon, (CAN)	Blue
Peralba	CO.NA.SE EU 63088 del 06/02/2023	White

Table.S2 Arabinoxylan, amylose, and β-glucan content.

Genotype	% WE_AX	% Tot Ax	% Amylose	β-glucan
Purendo	3.43 ± 0.30	49.14 ± 2.04	30.57 ± 2.09	0.52 ± 0.10
Vanil Noir	5.67 ± 0.33	46.76 ± 0.43	32.30 ± 1.19	0.40 ± 0.09
Peralba	4.52 ± 0.39	53.38 ± 0.42	31.89 ± 0.91	0.52 ± 0.04

A One-way ANOVA analysis was performed for each column, followed by Tukey's multiple comparisons post hoc test. No significant differences were observed among genotypes for any of the analyzed variables.

Table. S3 Physicochemical and Rheological Properties of whole flour.

Genotype	Moisture (%)	Protein (%)	Test weight (%)	Hardness %	SDS_test
Purendo	11.4 ± 0.05	16.0 ± 0.00^{a}	80.0 ± 0.05^{b}	146.7 ± 1.75^{a}	11.6 ± 0.3^{a}
Vanil Noir	11.6 ± 0.01	16.2 ± 0.15^{a}	79.8 ± 0.10^{b}	107.8 ± 0.60^{b}	12.55 ± 0.15^{a}
Peralba	11.3 ± 0.00	11.2 ± 0.00^{b}	84.9 ± 0.25^{a}	68.7 ± 0.25^{c}	9.05 ± 0.05^{b}

A One-way ANOVA analysis was performed for each column, followed by Tukey's multiple comparisons post hoc test. Values followed by a different letter are significantly different ($P < 0.01$).

Table S4. Evaluation of Color Attributes and Browning Index in Wheat Flours and Fractions.

Genotype	L*	a*	b*	100 - L*
Peralba Flour	91.10	-1.51	9.93	8.90
Peralba F250	84.66	0.27	9.21	15.34
Peralba G250	73.19	3.52	14.87	26.81
Vanilnoir Flour	86.73	-1.22	15.85	13.27
Vanilnoir F250	78.26	1.47	11.85	21.74
Vanilnoir G250	70.38	1.84	18.81	29.61
Purendo Flour	88.44	-1.85	5.64	11.56
Purendo F250	81.51	-0.69	5.23	18.49
Purendo G250	57.32	0.836	8.49	42.68

L*: Lightness — ranges from 0 (black) to 100 (white); indicates the brightness of the sample.
a*: Red/Green Index — positive values indicate red tones; negative values indicate green tones.
b*: Yellow/Blue Index — positive values indicate yellow tones; negative values indicate blue tones.
100 - L*: Browning Index — an inverse lightness value; higher values indicate darker (more browned) samples
